# Detecting and Quantifying Biomolecular Interactions of a Dendritic Polyglycerol Sulfate Nanoparticle Using Fluorescence Lifetime Measurements

**DOI:** 10.3390/molecules21010022

**Published:** 2015-12-24

**Authors:** Alexander Boreham, Jens Pikkemaat, Pierre Volz, Robert Brodwolf, Christian Kuehne, Kai Licha, Rainer Haag, Jens Dernedde, Ulrike Alexiev

**Affiliations:** 1 Institut für Experimentalphysik, Freie Universität Berlin, Arnimallee 14, 14195 Berlin, Germany; alexander.boreham@fu-berlin.de (A.B.); jpikkemaat@zedat.fu-berlin.de (J.P.); pierre.volz@fu-berlin.de (P.V.); brodwolf@zedat.fu-berlin.de (R.B.); 2 Helmholtz Virtual Institute—Multifunctional Biomaterials for Medicine, Helmholtz-Zentrum Geesthacht, Kantstr. 55, 14513 Teltow, Germany; haag@chemie.fu-berlin.de (R.H.); jens.dernedde@charite.de (J.D.); 3 Institut für Laboratoriumsmedizin, Klinische Chemie und Pathobiochemie, Charité—Universitätsmedizin Berlin, Augustenburger Platz 1, 13353 Berlin, Germany; christian.Kuehne@charite.de; 4 Mivenion GmbH, Robert-Koch-Platz 4, 10115 Berlin, Germany; Licha@mivenion.com; 5 Institut für Chemie und Biochemie, Freie Universität Berlin, Takustrasse 3, 14195 Berlin, Germany

**Keywords:** nanomedicine, dendritic polymers, protein corona, fluorescence lifetime

## Abstract

Interactions of nanoparticles with biomaterials determine the biological activity that is key for the physiological response. Dendritic polyglycerol sulfates (dPGS) were found recently to act as an inhibitor of inflammation by blocking selectins. Systemic application of dPGS would present this nanoparticle to various biological molecules that rapidly adsorb to the nanoparticle surface or lead to adsorption of the nanoparticle to cellular structures such as lipid membranes. In the past, fluorescence lifetime measurements of fluorescently tagged nanoparticles at a molecular and cellular/tissue level have been proven to reveal valuable information on the local nanoparticle environment via characteristic fluorescent lifetime signatures of the nanoparticle bound dye. Here, we established fluorescence lifetime measurements as a tool to determine the binding affinity to fluorescently tagged dPGS (dPGS-ICC; ICC: indocarbocyanine). The binding to a cell adhesion molecule (L-selectin) and a human complement protein (C1q) to dPGS-ICC was evaluated by the concentration dependent change in the unique fluorescence lifetime signature of dPGS-ICC. The apparent binding affinity was found to be in the nanomolar range for both proteins (L-selectin: 87 ± 4 nM and C1q: 42 ± 12 nM). Furthermore, the effect of human serum on the unique fluorescence lifetime signature of dPGS-ICC was measured and found to be different from the interactions with the two proteins and lipid membranes. A comparison between the unique lifetime signatures of dPGS-ICC in different biological environments shows that fluorescence lifetime measurements of unique dPGS-ICC fluorescence lifetime signatures are a versatile tool to probe the microenvironment of dPGS in cells and tissue.

## 1. Introduction

The molecular basis of nanoparticle interactions with cells and tissue can only be understood with precise knowledge of molecular properties of the nanoparticle. This characterization is of paramount importance, especially with regard to the targeted delivery of drugs or the physiological action of the nanoparticle itself. New and different analytical methods have to be established to meet the needs of the rapidly growing field of nanomedicine [1,2,3,4].

**Figure 1:**
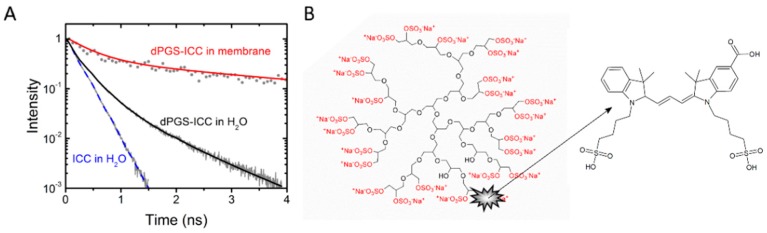
Structure and fluorescence lifetime characteristics of dPGS-ICC. (**A**) Fluorescence lifetime curves of ICC and dPGS-ICC in water and bound to DMPC lipid membranes; and (**B**) structural scheme of dPGS and the bound fluorescent tag ICC. A detailed description of the conjugation procedure is given by Licha *et al.* [13].

Important properties of dendrimeric nanoparticles like dendritic polyglycerol sulfates (dPGS) [5,6,7,8,9,10] include the size, shape and flexibility of the nanoparticle [11]. In a recent report we showed that both size and conformational flexibility of dPGS depend on temperature [1]. The fluorescent indocarbocyanine (ICC) (Figure 1) tag proved to be an efficient sensor for environmental properties as shown by the different fluorescence lifetimes in a systematic study of dPGS-ICC in different aqueous and organic solvents [1]. In addition to being dependent on the polarity [3], the fluorescence lifetime of the ICC dye is also sensitive to steric restrictions (Figure 1). In aqueous environments the ICC methine linker can rotate freely, leading to the short fluorescence lifetime of about 0.15 ns of the fluorophore. Steric hindrance of the ICC methine linker results in longer lifetimes [1,2,3,12]. A longer lifetime component of about 1 ns was observed for ICC bound to dPGS [2] (Figure 1A, Table 1). Upon binding of dPGS-ICC to lipid membranes an additional 4 ns component becomes apparent [1] (Figure 1A).

Upon administration of dPGS, the nanoparticle would interact with proteins of the blood serum or constituents of biological membranes (e.g., proteins and/or lipids). Interaction of nanoparticles with proteins is known as the “protein corona” [4,14] that determines the biological activity of the respective nanoparticle, because proteins compete with the target structures for the nanoparticle surface [15,16]. In the past several different methods were implemented to study the equilibrium and kinetic parameters of protein-nanoparticle interactions such as isothermal titration calorimetry (ITC), gel filtration, size-exclusion chromatography, surface plasmon resonance (SPR), or centrifugation based pull-down assays [4]. We recently established time-resolved fluorescence spectroscopy and fluorescence lifetime imaging microscopy (FLIM) as a versatile tool to analyze nanoparticle interactions at the molecular and cell/tissue level [1,2,3].

Here, we extended our previous fluorescence lifetime studies on dPGS-ICC to nanoparticle-protein interactions using L-selectin and the complement protein C1q as well as human serum. We show that fluorescence lifetime methods are well suited to detect the “protein corona” and to quantify binding affinities of individual proteins. Moreover, we show that different biomolecules display different fluorescence decay parameters underscoring our concept of using unique fluorescence lifetimes as target signatures in FLIM-based analyses of nanoparticle interactions in cells and tissue.

## 2. Results and Discussion

### 2.1. Determination of Apparent Protein Binding Constants for dPGS-ICC

#### 2.1.1. Binding of L-Selectin

We investigated fluorescence lifetime spectroscopy as a tool to determine affinity of protein–dPGS-ICC interaction. Multivalent dPGS is known to efficiently bind L- and P-selectin [6,7,9,10]. L-selectin is one of the natural cell adhesion molecules on leukocytes that in chronic inflammation processes extravasate into inflamed tissue by interactions of L-, E-, and P- selectins and their corresponding ligands consisting of fucosylated and sialylated glycoproteins on the endothelium.

**Figure 2:**
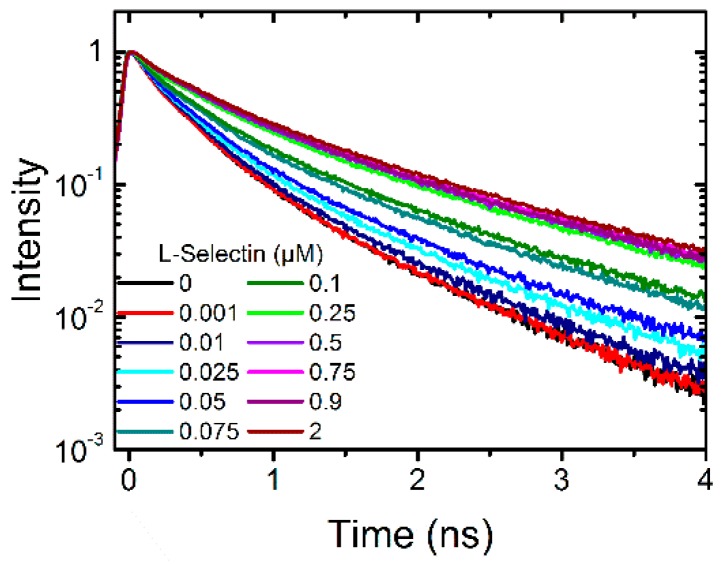
Fluorescence lifetime curves of 0.1 µM dPGS-ICC alone and with 11 different concentrations of soluble L-selectin-IgG chimera (0.001 µM to 2 µM) in DPBS at 20 °C.

First, soluble L-selectin (L-selectin-IgG chimera) was titrated in three different concentrations into a solution of 0.1 µM dPGS to evaluate whether a change in the fluorescence lifetime curve occurs. As this was the case we titrated 11 different concentrations into the dPGS-ICC solution until no change in the fluorescence lifetime curve was observable (Figure 2).

The fluorescence decay curves were fitted with a multi-exponential decay function and the mean fluorescence lifetime was determined. The time-resolved fluorescence method belongs to the indirect methods to determine a binding constant. The assumption is made that the measured fluorescence lifetime (or better the change upon binding) is directly proportional to the concentration of the nanoparticle-bound protein, assuming that the protein exists only in two states, in the nanoparticle bound and in the free state, each state having its own unique fluorescence lifetime characteristics.

**Figure 3:**
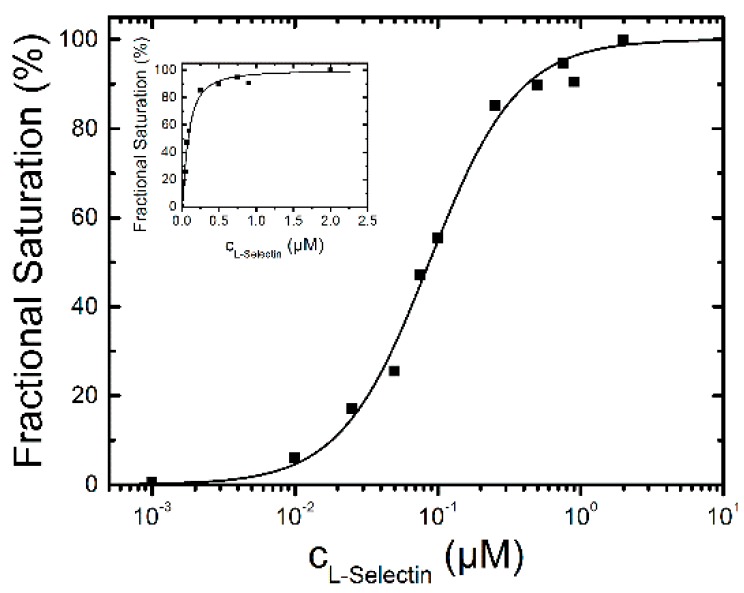
L-selectin-binding curve to 0.1 µM dPGS-ICC in DPBS at 20 °C. The concentrations are plotted on a logarithmic scale. The inset shows the data on a linear scale. The half maximum binding concentration was determined to be 87 ± 4 nM with a Hill-coefficient of *n* = 1.4.

To analyze the binding affinity, the difference between the mean fluorescence lifetime at 0 µM L-selectin and the mean fluorescence lifetimes at different L-selectin concentrations was calculated. The lifetime difference at 2 µM L-selectin constitutes the value at saturation of the binding reaction and was set as 100%. To determine the binding affinity the fractional saturation values (lifetime differences in %) were plotted *vs.* the concentration of L-selectin and fitted by a sigmoidal function (Figure 3). The half-maximum concentration of L-selectin binding was determined to 87 ± 4 nM with a Hill coefficient of *n* = 1.4 indicating a binding stoichiometry of about 1:1. The fluorescence decay time constants of the saturated lifetime signal are summarized in Table 1.

#### 2.1.2. Binding of C1q

**Figure 4:**
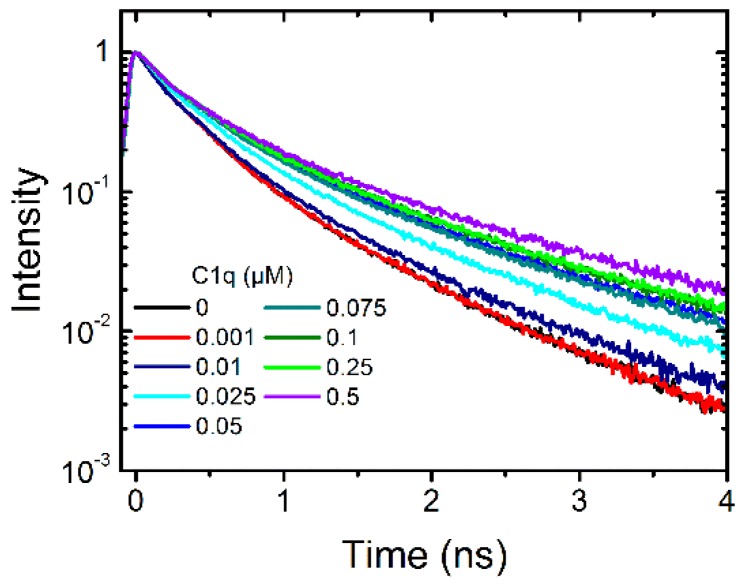
Fluorescence lifetime curves of 0.1 µM dPGS-ICC alone and with eight different concentrations of the complement protein C1q (0.001 µM to 0.5 µM) in DPBS at 20 °C.

Second, the complement protein C1q is the first protein that binds to immobilized antibodies and activates the classical pathway. In addition to its beneficial role in foreign antigen targeting, C1q plays an important role in autoimmune diseases and triggers inflammation. To study the interaction with dendritic polyglycerol sulfate C1q was titrated to a solution of 0.1 µM dPGS to evaluate whether a change in the fluorescence lifetime curve occurs. This was the case and we titrated eight different concentrations into the dPGS-ICC solution (Figure 4).

**Figure 5:**
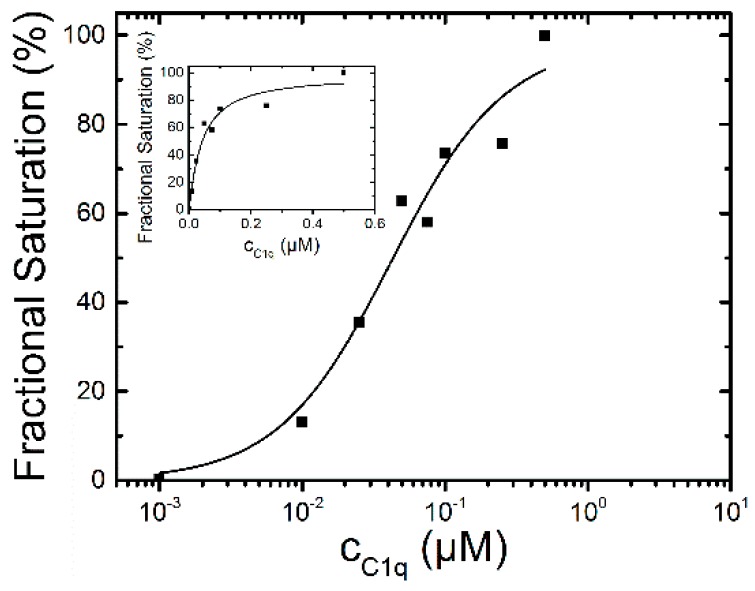
C1q- binding curve to 0.1 µM dPGS-ICC in DPBS at 20 °C. The half maximum binding concentration was determined to be 42 ± 12 nM and *n* = 1.1.

Using the fractional saturation method the apparent binding affinity was determined to be 42 ± 12 nM for C1q (Figure 5). The Hill coefficient was *n* = 1.1 indicating also for C1q a 1:1 binding stoichiometry. The fluorescence decay time constants of the saturated lifetime signal are summarized in Table 1.

#### 2.1.3. Binding of Human Serum

**Figure 6:**
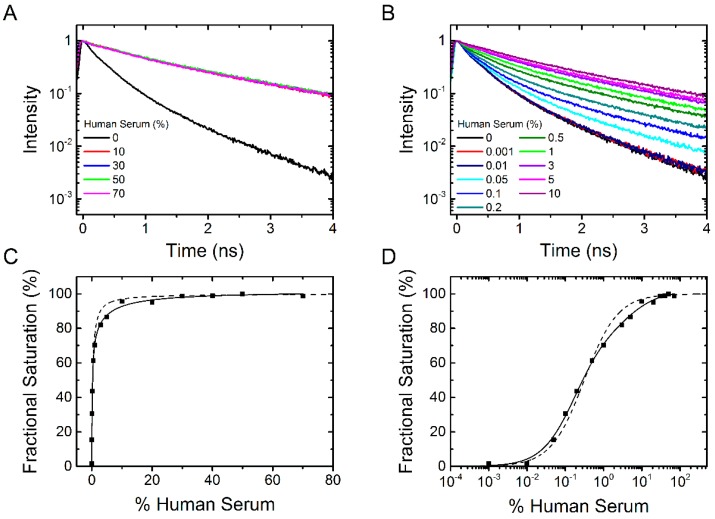
dPGS-ICC binding to human serum. (**A**) Fluorescence lifetime curves of 0.1 µM dPGS-ICC alone and with 10%, 30%, 50%, and 70% human serum in DPBS at 20 °C; (**B**) Fluorescence decay curves for titration of dPGS-ICC with human serum at different concentrations between 0.001% and 10% serum; (**C**,**D**) Fractional saturation as a function of human serum concentration both linear (**C**) and with a logarithmic *x*-axis (**D**). The half maximum binding concentration was determined to be 0.3% human serum (using a Hill-coefficient of *n* = 1) for a single component fit and 0.2% and 5% for a double component fit.

Next we tested at which concentration (in %) human serum with a protein content of 53 mg/mL results in a saturation of the fluorescence lifetime signal. Figure 6A shows the fluorescence lifetime curves of dPGS-ICC with 0%, 10%, 30%, 50%, and 70% serum concentration. These data clearly show that already at about 10% serum concentration (equivalent to a 5.3 mg/mL solution) the saturation fluorescence signal is reached. The fluorescence decay time constants of the saturated lifetime signal are summarized in Table 1.

To determine the binding affinity of human serum constituents to dPGS we titrated human serum at different concentrations between 0.001% and 10% serum (Figure 6B). Using the fractional saturation method, the mean fluorescence lifetime derived data were plotted in percent as a function of human serum concentration in Figure 6C,D. Analysis of the binding curve gives a mean apparent binding affinity corresponding to 0.3% serum (with a Hill coefficient *n* = 1). In contrast to the plots shown in Figure 3 and Figure 5 the sigmoidal fit (Figure 6D) does not completely fit the data. This is understandable as the serum consists of a mixture of different proteins which could bind with different affinities. We thus added a second component to the fit and the resulting apparent binding affinities (half maximum binding concentration) were 0.2% serum (78% amplitude) and 5% serum (22% amplitude). Even though there are more than two different proteins in the human serum, the fit considerably improves. We conclude that dPGS has the potential to bind different protein species with varying binding affinity. This is an important result as it provides a rationale that, despite its protein corona, dPGS recognizes L-selectin in living tissue [6].

#### 2.1.4. SPR Measurements of Protein Association

**Figure 7:**
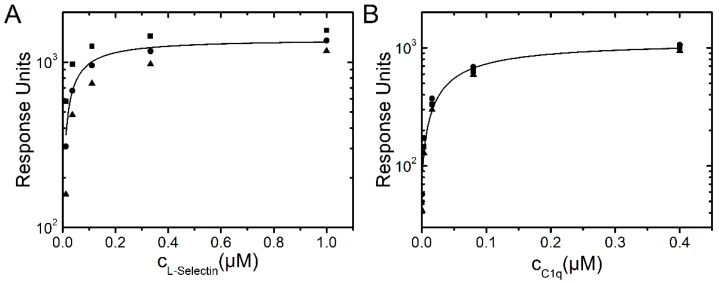
Langmuir binding isotherms of L-selectin (**A**) and C1q (**B**) to dPGS.

L-selectin binding to dPGS was also investigated by surface plasmon resonance (SPR). The dPGS was immobilized on a chip and the defined concentrations of the analytes L-selectin and C1q were passed over the functionalized surface. Binding affinity was determined from resulting binding isotherms (Figure 7) and gave values of 45 ± 17 nM for L-selectin and 62 ± 10 nM for C1q. These complementary experiments prove that binding of L-selectin and C1q to dPGS determined by either analysis of fluorescence decay curves or SPR revealed comparable affinities in the lower nanomolar range.

### 2.2. Determination of Protein Binding Kinetics to dPGS-ICC

#### 2.2.1. Binding Kinetics of Human Serum

**Figure 8:**
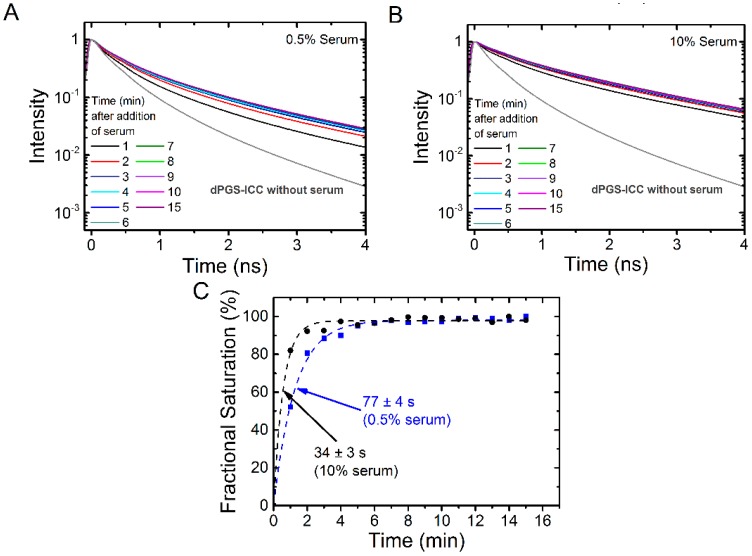
Kinetics of dPGS-ICC binding in human serum. Lifetime decay curves in (**A**) 0.5% serum and (**B**) in 10% human serum at different time points are shown; (**C**) Fractional saturation, based on the mean fluorescence lifetimes, as a function of time. The dashed lines indicate an exponential fit to the data and the time constant τ was 34 s for 10% serum and 77 s for 0.5% serum.

Binding kinetics measurements offer an additional approach to evaluate protein association. We first tested the binding kinetics of different concentrations of human serum. We choose two concentrations, near the half maximum binding concentration (0.5% serum, Figure 8A) and a concentration that leads to saturation of the fluorescence lifetime signal (10% serum, Figure 8B). Figure 8C shows the binding curves. As expected from the fractional binding signal (Figure 6), the kinetics is faster for 10% serum (34 ± 3 s) than for 0.5% serum (77 ± 4 s).

#### 2.2.2. Binding Kinetics of L-Selectin

**Figure 9:**
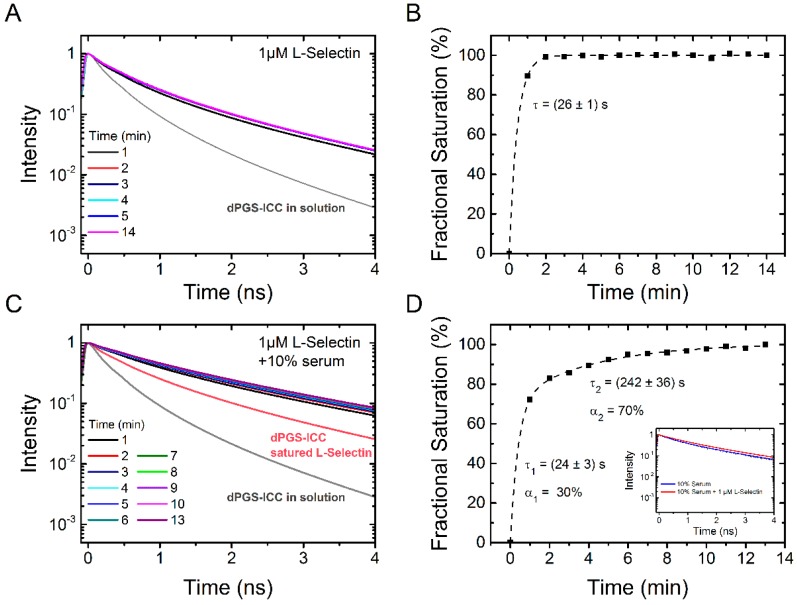
Time dependence of dPGS-ICC binding to L-selectin and effect of serum. (**A**) Lifetime decay curves of 0.1 µM dPGS-ICC binding to 1 µM L-selectin and (**B**) the fractional saturation, based on the mean fluorescence lifetimes, as a function of time. The value of τ = 26 s was obtained from an exponential fit to the data (dashed line); (**C**) Lifetime decay curves of 0.1 µM dPGS-ICC binding to 1 µM L-selectin upon addition of human serum (final concentration: 10% serum) and (**D**) the fractional saturation, based on the mean fluorescence lifetimes, as a function of time. A bi-exponential fit to the data (dashed line) yielded two time constants of τ_1_ = 24 s and τ_2_ = 242 s with amplitudes of 30% and 70% respectively. Inset: fluorescence lifetime curves of dPGS-ICC in 10% serum and in 10% serum with 1 µM L-selectin.

Next, we determined the binding kinetics of L-selectin (Figure 9A,B). At the saturation concentration of 1 µM, L-selectin binds very fast with a time constant of 26 ± 1 s. This is in the same range as the binding time constant of 10% serum (Figure 8). Subsequent addition of 10% serum to L-selectin saturated dPGS-ICC leads to further increase of the fluorescence lifetime (Figure 9C). Evaluation of the kinetics of serum binding to L-selectin saturated dPGS-ICC reveals a bi-exponential binding kinetics with time constants of 24 ± 3 s (30% amplitude) and 242 ± 36 s (70% amplitude). This is in contrast to the monoexponential binding kinetics with a time constant of 34 s observed for 10% serum alone. Clearly, L-selectin competes with the binding of other serum proteins. A comparison of the unique lifetime signatures of dPGS-ICC with 10% serum and dPGS with L-selectin/10% serum shows subtle but clear differences that indicate the presence of dPGS bound L-selectin under saturating binding concentrations of human blood serum. This result supports our conclusion from the previous section that dPGS recognizes L-selectin despite the presence of other competing proteins in blood plasma.

**Table 1:** Fluorescence lifetime parameters for different dPGS-ICC interaction partners according to Equation (1). The mean fluorescence lifetime (Equation (2)) and the reduced Χ^2^ is given.

Sample	α_1_ (%)	τ_1_ (ns)	α_2_ (%)	τ_2_ (ns)	α_3_ (%)	τ_3_ (ns)	τ_mean_ (ns)	Χ^2^_R_
0.1 µM dPGS-ICC	5.7	1.16	30.7	0.47	63.6	0.17	0.51	1.05
+ 0.5 µM C1q	11.2	1.85	31.3	0.67	57.5	0.18	1.04	0.95
+ 1 µM L-selectin	17.4	1.75	40.5	0.65	42.1	0.19	1.11	1.01
+ 1 µM L-selectin + 10% Serum	37.1	2.24	39.5	0.90	23.4	0.19	1.78	0.91
+ 70% Serum	39.2	2.27	35.1	0.85	25.7	0.14	1.87	0.98

## 3. Materials and Methods

### 3.1. Time-Resolved Fluorescence Setup

All measurements were performed on a time-resolved fluorescence setup [17,18,19] with a tunable white light laser source (SuperK Extreme EUV3, NKT, Birkerød, Denmark), a microchannel plate detector and time-correlated single photon counting (TCSPC) electronics (SPC-830, Becker & Hickl GmbH, Berlin, Germany) with picosecond time resolution. The excitation of ICC at 530 nm was selected via an acousto-optical tunable filter (SELECT UV-VIS, NKT, Birkerød, Denmark). Fluorescence emission was collected above 545 nm using a long-pass filter (HQ545 LP, Chroma, Bellows Falls, VT, USA). The excitation power was 220 µW and the repetition rate was 19.5 MHz. The time range was set to 10 ns divided into 1024 channels resulting in a resolution of 9.8 ps/channel. The instrument response function of the system was ~54 ps full width at half maximum. 

### 3.2. Experimental Procedures

#### 3.2.1. Dendritic Polyglycerol Sulfate Nanocarrier Labeled with a Fluorescent ICC Dye (dPGS-ICC)

Dendritic polyglycerolsulfate (dPGS) with a molecular weight of approx. 12,000 g/mol was synthesized according to literature via an anionic multi-branching ring-opening polymerization of glycidol and sulfation using sulfurtrioxide-pyridinium complex [20]. A detailed description of the dye conjugation procedure is given by Licha *et al.* [13]. Briefly, to attach ICC to dPGS, an azido-linker (linker 11-azido-1-undecanyl-tosylate) was conjugated to the polyglycerol scaffold at a molar ratio of approximately one linker per polymer before sulfation. After sulfation, the azido-containing polymer was conjugated with a propargyl derivative of ICC by copper-catalyzed 1,3-dipolar cycloaddition (click conjugation) in water/ethanol. After synthesis, the dPGS-ICC was lyophilized. All experiments were conducted with a freshly prepared dPGS-ICC sample from the lyophilisate of the same batch.

#### 3.2.2. Determination of Apparent Protein Binding Constants for dPGS-ICC

To determine the apparent protein binding constants for dPGS-ICC, samples containing 0.1 µM dPGS-ICC and varying concentrations of either L-selectin (L-selectin-IgG chimera, R & D Systems, Wiesbaden, Germany) or C1q (Fitzgerald Industries International, Acton, MA, USA) were prepared in DPBS buffer (PAA; 10 mM phosphate, 1 mM calcium, 1 mM magnesium, 3 mM KCl, 137 mM NaCl, pH 7.4). The measurements were conducted after incubating 0.1 µM dPGS-ICC and the different protein concentrations for 2 min at room temperature. In addition the binding behavior of dPGS-ICC in human serum was analyzed. Here, fluorescence lifetime measurements were conducted after incubating 0.1 µM dPGS-ICC and different concentrations of serum for 5 min at room temperature. The fluorescence decay was recorded for 180 s.

#### 3.2.3. Determination of Protein Binding Kinetics to dPGS-ICC

To determine the kinetics of dPGS-ICC binding in human serum, a buffer solution containing dPGS-ICC was prepared. Directly before the start of the measurement human serum was added to either 10% or 0.5% final concentration. The concentration of dPGS-ICC in the final volume was 0.1 µM in both cases. The fluorescence decay was recorded over a time period of 15 min, with a duration of 60 s for each individual recording.

#### 3.2.4. Determination of Protein Exchange

To determine the influence of human serum on preincubated L-selectin, first the kinetics of dPGS-ICC binding to L-selectin were determined and then the effect of human serum addition was followed. A buffer solution containing dPGS-ICC was prepared. Directly before the start of the measurement L-selectin was added at a concentration of 1 µM. The concentration of dPGS-ICC in the final volume was 0.1 µM. The fluorescence decay was recorded over a time period of 14 min, with a duration of 60 s for each individual recording. Then, human serum was added, final concentration 10%, and further measurements were conducted for the next 13 min, again with a duration of 60 s for each individual recording.

#### 3.2.5. SPR Measurements of Protein Association

Experiments were carried out on a Biacore X100 device (GE Healthcare, Freiburg, Germany). A streptavidin coated chip (SA-Chip, GE Healthcare) was coupled with dPGS-biotin to a level of ~400 resonance units (RU) in HBS-Ca (10mM HEPES pH 7.4 + 150 mM NaCl + 1 mM CaCl_2_) as a running buffer using standard procedures. Briefly, the chip was conditioned using three consecutive injections of 60 s 1 M NaCl + 50 mM NaOH before injection of dPGS-biotin and washing with three consecutive injections of running buffer. Affinities were measured using a kinetic titration series (single cycle kinetics) at 25 °C in which five ascending concentrations of the analyte (C1q or L-Sel-IgG chimera) were injected consecutively for 120 s at 30 µL/min followed by a dissociation time of 600 s and one regeneration step with 4 M MgCl_2_ for 120 s. L-selectin was diluted in HBS-Ca to final concentrations of 1000, 333, 111, 37, and 12.34 nM; C1q in surfactant P20 supplemented (+0.005%) running buffer (HBSP) at concentrations of 400, 80, 16, 3.2, and 0.64 nM. The signal of the untreated flow cell was subtracted from the binding signal. Additionally, blank injects of running buffer only were also subtracted (double referencing) for each run. Sensorgrams were analyzed by plotting the analyte concentration against the binding signal at the end of inject. The resulting isotherm was fitted using the steady state model.

### 3.3. Data Analysis

#### 3.3.1. Determination of Fluorescence Decay Parameters

The fluorescence decay profiles were analyzed using the software package Globals Unlimited V2.2 (Laboratory for Fluorescence Dynamics). An algorithm based on a Marquardt–Levenberg type of nonlinear least-squares analysis was used. The time course of the fluorescence was fitted with a sum of exponentials:

(1) $$I\left(t\right)=\underset{i}{\sum }a_{i}*\exp\left(-\frac{t}{\tau_{i}}\right)$$

where *a_i_* are the amplitudes and τ*_i_* are the lifetimes of the *i*th decay component. α*_i_* are the corresponding relative amplitudes, with α*_i_* = *a_i_*/∑*a_i_* [17,21].

#### 3.3.2. Determination of Apparent Binding Constants

To determine the apparent binding constants of dPGS-ICC, the mean lifetime of the respective fluorescence decay curves was calculated as follows:

(2) $$\tau_{mean}=\underset{i}{\sum }\tau_{i}*\left(\frac{\alpha_{i}*\tau_{i}}{\sum_{i}\alpha_{i}*\tau_{i}}\right)$$

where α*_i_* are the relative amplitudes and τ*_i_* are the lifetimes of the *i*th decay component.

The fractional saturation (in %) was determined as follows:

(3) $$fractional\;saturation\;\left(\%\right)=\left(\frac{\tau_{mean}-\tau_{0}}{\tau_{max}}\right)*100$$

where τ*_mean_* is the mean lifetime of the respective lifetime decay curve, τ_0_ is the mean lifetime of the lifetime decay curve of dPGS-ICC in solution, and τ*_max_* is the highest mean lifetime.

The resulting data points were then fit according to the model function

(4) $$y\left(x\right)=\frac{S*x^{n}}{\left({K_{50}}^{n}+x^{n}\right)}$$

where *S* is the saturation of binding, *K*_50_ is the half maximum binding concentration (apparent binding affinity), and the Hill-coefficient *n*, a cooperativity factor.

#### 3.3.3. Determination of Protein Binding Kinetics to dPGS-ICC

To determine the binding kinetics of dPGS-ICC, the mean lifetime of the respective fluorescence decay curves was calculated as described in Equation (2) and the fractional saturation (in %) was calculated according to Equation (3). The kinetic data was then fit with an exponential model function.

(5) $$y\left(t\right)=A*e^{\frac{t}{\tau }}$$

#### 3.3.4. Determination of Protein Exchange

The binding kinetics of dPGS-ICC to L-selectin were determined as described in 3.3.2. To evaluate the kinetics of serum addition the fractional saturation (in %) was determined using Equation (3), however with τ_0_ the mean lifetime of the lifetime decay curve of dPGS-ICC bound to L-selectin. The kinetic data required a fit with a biexponential model function according to:

(6) $$y\left(t\right)=A_{1}*e^{\frac{t}{\tau_{1}}}+A_{2}*e^{\frac{t}{\tau_{2}}}$$

## 4. Conclusions

**Figure 10:**
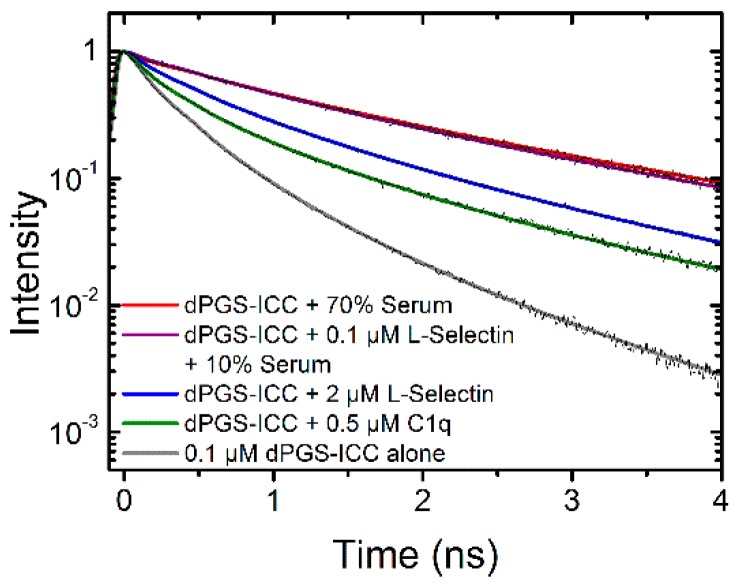
Lifetime signatures of dPGS-ICC with different proteins and human serum.

dPGS is a multivalent dendritic negatively-charged nanoparticle whose binding affinities are largely determined by electrostatic interactions. For example binding to L-selectin occurs via a patch of basic amino acid residues in the lectin binding domain [22]. However, other proteins including membrane proteins very often feature charged patches that may also undergo transient changes upon protein function [23,24,25]. Thus it is very likely that in a physiological environment a plethora of dPGS binding partners exists. Here, a comparison of the lifetime signatures of dPGS-ICC with different proteins present in human serum and cellular membranes, like C1q and L-selectin, respectively, shows clear differences (Figure 10).

Previously-published data on dPGS-ICC showed that the two fastest decay components reflect on the polarity of the ICC environment while the slowest component is due to steric hindrance of the methine linker rotation caused by the PG branches [1]. Here, we observed that both the slowest component and the intermediate component show slower lifetime values upon dPGS-ICC binding to the proteins L-selectin and C1q but also in the presence of human serum, with the exact values depending on the specific protein. This clearly indicates that the binding of dPGS-ICC to the proteins L-selectin and C1q, but also to binding partners in human serum, changes both the polarity of the immediate dPGS-ICC environment and also the steric hindrance for the rotation of the ICC methine linker in a protein dependent fashion.

The measurements presented in this paper were performed in solution. However, fluorescence lifetime can also be recorded in a spatial resolved fashion on cells and tissues under a microscope, *i.e.,* with fluorescence lifetime imaging microscopy (FLIM). The use of FLIM is known to include environmental sensing of, amongst others, polarity, local pH, and calcium concentrations, as well as the study of protein interactions in living cells [26]. The results provided in this study show that the fluorescence lifetime also allows for the environmental sensing of biomolecular interactions with dPGS, as unique dPGS-ICC lifetimes exist depending on the dPGS binding partner (Figure 10). Thus, we now extend our concept of using unique fluorescence lifetime signatures for fast and reliable localization of fluorescently labeled nanoparticles in cellular systems and tissue samples [2,27,28,29,30] to the potential detection of biomolecular interactions of dPGS in physiological environments. The results presented allow for the possibility of specifically determining the dPGS interaction partners based on the specific fluorescence signature. Further, this methodology is not necessarily limited to dPGS, as in theory any nanoparticle with a fluorescent reporter group can be used, as long as the environmental sensitivity is high enough. However, the use of ICC, as in this study, offers the additional benefit of being sensitive to the steric hindrance of the dye upon nanoparticle-biomolecule interactions. This concept will be developed further in future experiments.

## Abbreviations

The following abbreviations are used in this manuscript:

dPGS	Dendritic polyglycerol sulfate
dPGS-ICC	Indocarbocyanine bound to dendritic polyglycerol sulfate
ICC	Indocarbocyanine
DMPC	Dimyrostoylphosphatidylcholine
ITC	Isothermal titration calorimetry
FLIM	Fluorescence lifetime imaging microscopy
TCSPC	Time-correlated single photon counting
IgG	Immunglobulin G
RU	Resonance units
SPR	Surface plasmon resonance
